# Annotations

**Published:** 1899-01-28

**Authors:** 


### ANNOTATIONS.

### The Inebriates Act.

WITH the passing of the Criminal Inebriates Act a revolution in the treatment of inebriety has taken place, and one which will commend itself to every one but those unreasoning agitators (misnamed "temperance") who refuse to see that many—thank Heaven, the majority of people—can take a small quantity of alcohol daily during the whole of a long and blameless life without being hurt thereby; while others, truly victims of disease, cannot taste alcohol without becoming the victims of drunkenness. The treating of alcoholism as a crime has been long shown to be a failure. To imprison a drunkard for a few days just gives him time to get over the nausea of the last bout, and sends him out to the world ready for another one. The repeated appearances of some drunkards in our Courts show this, while they also give the impression that we are more of a nation of drunkards than is the case. A hundred convictions for drunkenness in a town may be all due to the presence of some half-dozen drunkards; and it is just as well to clear the national reputation from this slander. It is probable that the cost of keeping confirmed drunkards in reformatories for a time sufficient to give them a chance of being cured of their disease will not be so great as some people think. Many of those who will be affected by the new Act pass a great part of their life in prison anyway, and the cost of continually arresting and convicting them will be saved.

### A Race Against Death.

THE difficulty of doing good without at the same time causing evil has lately received a curious illustration in New York. Everyone has heard of the marvellous rapidity with which the firemen in that city turn out on receiving a "call," and a system on somewhat the same lines has been devised for the conveyance of first aid in case of accident. The ambulance arrangements in New York have been carried to a pitch of perfection which is almost inconceivable to those who are only accustomed to the "go as you please" arrangements in this country. And all this is very good. What, however, has lately scandalised the people of New York is the discovery—which in so 'cute a people seems to us to have been made rather late—that, even in well-doing, the prospect of a glass of beer is a stimulus to still greater efforts. This has long been known in London, and we fancy that in those parts which happen to lie fairly equidistant between several hospitals the comparative merits of the various taps are pretty well known to those mostly concerned in picking up accidents from our streets. Of course, these things are not done officially. It is the enthusiasm of youth, of house surgeons and of dressers anxious to get plenty of good cases that is at the bottom of the affair; and it is, we fancy, a pretty heavy tax on some of them. In New York, however, where the different hospitals fetch in their own accidents by means of special ambulances always waiting on the pounce for cases, it must sometimes happen that a little delay may lead to dead men being brought in; and hence has arisen the ingenious plan, which is now said to have taken the form of an unwritten law of the ambulance service, according to which any surgeon bringing in a corpse shall pay for a keg of beer. That keg of beer is at the bottom of all the trouble. We have no doubt ourselves that by its aid New York has a much smarter ambulance service than it would have without it; but all the same, it is said that, while the hope of beer is a stimulus to the drivers, the fear of having to provide it makes the surgeons in charge rush their patients. We do not think that the good people need agitate themselves much about it. We must remember that the whole of this ambulance system goes on the theory that immediate removal is the best plan, the great excuse for the elaborate arrangements in force being that patients should be got into hospital as quickly as possible. No doubt such a race with death, with beer as both penalty and reward, is not a very dignified proceeding, and we would gladly see an end put to all such aids to activity, but we very much doubt whether any real harm has been done.

### Employment for the Infirm.

A GREAT deal of spurious indignation has been expended during the past few weeks over the discovery of a fact which we suppose could have been discovered at any time by anyone who wanted to find it out, namely, that the work done by workhouse inmates under the guidance and initiation of the Brabazon Society is sold for money, and that the money which it fetches goes neither to those who do the work nor to the workhouses in which it is done. None the less, the Brabazon Employment Society is a most beneficent institution. If we think what it is to be an aged or disabled pauper, cut off from friends and relatives, cut off from all chance of employment except such as is compulsory, and thus monotonous, cut off from all opportunity of exercising one's intelligence and one's ingenuity, and with nothing to look forward to but living out one's life as best one may—if we try to realise what this is, we can imagine what a boon it must be to these poor people to be given some interest in life again. No doubt it is the duty of the master of a workhouse to provide employment, but occupation set by the master is a task; while the aim of the Brabazon Society is to make the work a pleasure and an interest. Writing to the *Times*, the Duchess of Montrose says that no remuneration is given to any pauper for his labour, but that the proceeds from the annual sales are devoted to providing fresh material for work, and any surplus which is over is spent on a treat for the inmates of the workhouse, or on books, &c., for the wards. The majority of the poor people who do the "work" would be utterly unfit to do anything were it not for the patient teaching of the kind ladies who are Brabazon Society workers. Occupation is worth anything to the infirm, and nothing is more difficult than to find it for them without making it a task. The Duchess says: "I can testify that it has brightened the lives of hundreds of infirm paupers by diverting their minds from their own ailments and misfortunes and providing them with interesting occupation for the good of others." The curse of the workhouse for the aged and infirm is the aimlessness of the life, and so far as this is lightened by the Brabazon Employment Society its work is good.

